# Bad Business Methods

**Published:** 1901-08-15

**Authors:** 


### BAD BUSINESS METHODS.

The idea of some physicians and dentists that their accounts should not be rendered monthly or only when requested is a great mistake. It is mistaken charity and it is a business fault. A practitioner with a large practice may think it is his skill that has accomplished it. He has been ever exceedingly "easy" with his patrons, allowing accounts to run on from months into years. Too considerate in the first place and then too busy to push them. When pressed by his creditors his well-to-do and willing debtors being called on in the emergency to help him out of a tight place. Really it was and is this easy way of his that has attracted his following and keeps it to him, for the world generally believes one professional man just as good as another for ordinary purposes, and will employ the one whose services may be secured on the easiest terms. In large centers of population business managed this way may hurt only the doctor himself, but in small communities it is a decided detriment to himself and a great harm to his brother professional, whose credit he impairs with his own in consequence of the lack of ready money from a badly managed business. Again, when rushed to distraction with work, it naturally results that the same care and precision is not taken in operations. They fail, and the profession at large is condemned. We have in mind two such practitioners, one of the medical and the other of the dental profession. Both very clever and popular, who conscienciously think they are doing life's duty well, and yet they are wearing out themselves with hard work, partially paid for, their families stinted and their brother practitioners in no lovely mood toward them.—Editorial in *Dental Hints*.

